# Formation mechanism of insensitive tellurium hexanitride with armchair-like cyclo-N_6_ anions

**DOI:** 10.1038/s42004-020-0286-1

**Published:** 2020-04-02

**Authors:** Zhao Liu, Da Li, Quan Zhuang, Fubo Tian, Defang Duan, Fangfei Li, Tian Cui

**Affiliations:** 1 State Key Laboratory of Superhard Materials, Jilin University, Changchun, 130012 People’s Republic of China; 2 School of Physical Science and Technology, Ningbo University, Ningbo, 315211 People’s Republic of China

**Keywords:** Electronic properties and materials, Chemical bonding, Computational chemistry

## Abstract

The lower decomposition barriers of cyclo-N_6_ anions hinder their application as high-energy-density materials. Here, first-principles calculations and molecular dynamics simulations reveal that enhancing the covalent component of the interaction between cyclo-N_6_ anions and cations can effectively improve the stability of cyclo-N_6_ anions. Taking tellurium hexanitride as a representative, the exotic armchair-like N_6_ anions of tellurium hexanitride exhibit resistance towards electronic attack and gain extra stability through the formation of covalent bonds with the surrounding elemental tellurium under high pressures. These covalent bonds effectively improve the chemical barrier and insensitivity of tellurium hexanitride during blasting, which prevents the decomposition of solid cyclo-N_6_ salts into molecular nitrogen. Furthermore, the high-pressure induced covalent bonds between cyclo-N_6_ anions and tellurium enable the high bulk modulus, remarkable detonation performance, and high-temperature thermodynamic stability of tellurium hexanitride.

## Abstract (web summary)

The lower decomposition barriers of cyclo-N_6_ anions hinder their application as high-energy-density materials. Here, first-principles calculations reveal that enhancing the covalent component of the interaction between cyclo-N_6_ anions and cations can effectively improve stability at high pressure.

## Introduction

High pressure, a typically clean and controllable thermodynamic variable, can be adopted to obtain curious materials that are difficult to synthesize under ambient condition^1–3^. Moreover, the precompression evoked by metal elements can reduce the required external pressure for the synthesis of these materials^4^. Under high pressure, metal nitrides have attractive physical and chemical characteristics, such as good superconductivity, good magnetism, good hardness, and a particular catalytic performance^5–8^. Metallic nitrides are conducive to optoelectronic and defect-tolerance characteristics and have strong metal–nitrogen bonds for structural stability and mechanical stiffness^9^. Particularly, the compression of N-rich nitrides has been recommend as an alternative method to obtain metallic atomic nitrogen states as high-energy density materials since the laser-heated diamond anvil cell, which is a powerful tool, has been used to synthesize a series of stable monatomic forms of solid nitrogen^10–13^. Synthesizing pentazolate or *π*-aromatic ions is considered one of the best and most efficient methods to obtain metallic poly-nitrogen phases^14^. However, all *π*-aromatics are incredibly unstable, difficult to synthesize, and sensitive to electrophilic attack, and they mostly appear nonmetallic^15^.

Many attempts have been made to synthesize pentazolate anion until cyclo-N_5_^−^, and the first attempt was first reported in 1998^16^. Later, the pentazolate salt in solid (N_5_)_6_(H_3_O)_3_(NH_4_)_4_Cl was reported with a stable thermal decomposition temperature (390 K) via thermogravimetric experiments^17,18^. Recently, the controllable and synthetic cyclo-N_5_ ionic salt CsN_5_ was reported at 60 GPa with a high-energy density and a relatively assessable pressure^19^. Compressing CsN_3_ mixed with N_2_ cryogenic liquid was also used to achieve cyclo-N_5_ ionic salt according to a synchrotron X-ray diffraction measurement at 55.4 GPa in a diamond anvil cell. A Raman spectral vibration mode unique to the cyclo-N_5_^−^ anion was observed in LiN_5_ salt^20^. Because of the intrinsic stability of N_5_^−^ anions, their crystals have considerable kinetic stability that may be sufficient for an ambient pressure recovery. Considering their electronic structures, the effective separation of the *σ* and *π* electrons that correspond to the highest occupied molecular orbital (MO) and lowest unoccupied MO can help to stabilize cyclo-N_5_^−^/N_5_^+^ salt^21^. Likewise, the pursuit of energy-intensive cyclo-N_6_ salts with higher nitrogen contents than pentazolate anions, which are synthesized at a modest pressure, has never ceased^22^. However, the cyclo-N_6_ ionic salt only remains in the theoretical stage. Numerous planar or quasi-planar cyclo-N_6_ anions are predicted at high pressure in Li, Mg, Cs, Ca, Rb, and Ba nitrides, but they have not been successfully synthesized for unclear reasons^6,22–25^.

These theoretical studies did not pay close attention to the microstructural characteristics of cyclo-N_6_ anions. It is of note that an in-plane distortion may occur in cyclo-N_6_ sub-lattices when the neutral *π-*aromatic switches to a charged anion. For instance, armchair-like N_6_ rings are predicted in *h*-W^2.4+^N_6_^2.4− 26^. Once a benzene-like molecule forms a cyclo-N_6_^6−^ anion, its symmetry reduces and even decomposes because its antibonding states are fully occupied, which may prevent experimental synthesis. Another reason may be that the decomposition barrier of cyclo-N_6_ anions is extremely low and they spontaneously decompose to other anions. We propose a strategy to maintain the “non-molecular nitrogen phase”, i.e., to enhance its energy barrier and insensitivity via covalent bonds entrapment between metal/nonmetal and cyclo-N_6_ ions to keep it from breaking down into the molecular phase. Based on these judgments, armchair-like cyclo-N_6_ anions may be able to stabilized if their antibonding MOs are not completely occupied and their structures are entrapped by covalent effects. Considering that Te has higher electronegativity and a larger atomic radius than W, it easily forms covalent bonds with nitrogen; thus, we adopt the binary Te–N candidates as prototypes to search for cyclo-N_6_ ions and study the trap effect by the covalent bond^27,28^.

In this work, our broad structure searches combined with first-principles simulations identify a TeN_6_ nitride with armchair-like cyclo-N_6_ anions, high-pressure–temperature stability and remarkable mechanical properties. Herein, tellurium serves as an electron donor to modulate the electronic distribution and forms covalent bonds with nitrogen atoms, which induces metallic cyclo-N_6_ anions. Meanwhile, covalent bond entrapment to stabilize the armchair-like cyclo-N_6_ anions is revealed. More importantly, the covalent bonds effectively improve the chemical barrier and insensitivity to prevent the decomposition of monatomic forms of solid N_6_ anions into the molecular phase. Moreover, the detonation performance and energy density of the metallic cyclo-N_6_ anions predicted by our study are higher than those of most previously reported pentazolate and six-membered N_6_ anions in binary nitrides.

## Results and discussion

### Phase stability and structural features at high pressure

**Fig. 1:**
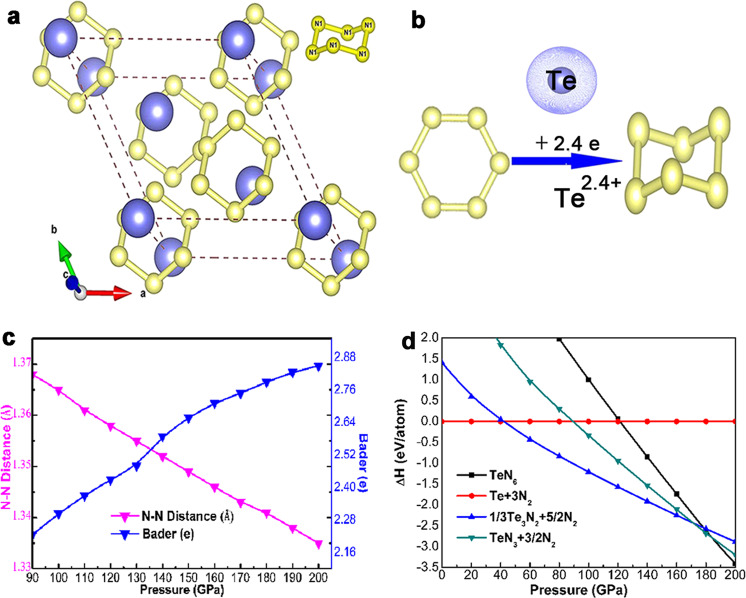
Structural properties and enthalpies of TeN_6_ under high pressures. **a** Stable structures of the anti-CdCl_2_ phase at 120 GPa. **b** Planar cyclo-N_6_ sub-lattice interacted with Te atoms to form armchair-like N_6_ anions. **c** N–N bond lengths and Bader charge transfer as a function of pressure in TeN_6_. **d** Enthalpy of the anti-CdCl_2_ phase relative to the mixture of *P*3_1_21-Te, TeN or TeN_3_ and the *P*4_1_2_1_2 nitrogen phases.

A neutral cyclo-N_6_ molecule with inherent benzene-like structure has planar D_6h_ symmetry^29^. However, the crystalline sub-lattice N_6_ isolated anions have D_3d_ symmetry in the anti-CdCl_2_ TeN_6_ phase (space group *R*-3*m*, Supplementary Tabel 1) because of structural mutations, as shown in Fig. 1a and Supplementary Fig. 1. The armchair-like structural configuration of the equivalent bonding in 3D space hints that the nitrogen atoms adopt *sp*^3^ hybridization to form *σ* covalent bonds. The distance between nitrogen atoms is 1.37 Å at million magnitude pressure, which is a prototypical N–N single bond without the resonance effect between alternating *π*- and σ-bonds. After removing tellurium atoms from the anti-CdCl_2_ phase, as shown in Fig. 1b, the cyclo-N_6_ structure transforms into a flat shape with D_6h_ symmetry but remains in the anti-CdCl_2_ phase. Compared with the anti-CdCl_2_-TeN_6_ phase, the volume of planar cyclo-N_6_ decreases by 20%, which suggests that the structural deformation of cyclo-N_6_ is related to the interactions with tellurium atoms. Thus, the N–N bond lengths, Bader charge transfer and volumes as functions of pressure are analyzed to gain insight into the interaction between atoms. The volume decreases by 0.20 Å^3^/GPa almost linearly with pressure, which indicates strong incompressibility, as shown in Supplementary Fig. 2, while the charge transfer amount gradually increases, as shown in Fig. 1c. However, the change in the N–N distances under high pressure is extremely weak, which preserves N–N single bonds. Herein, the decrease in lattice volume under pressure is mainly attributed to the Te–N distance shrinkage, which increases the interaction and stimulates new physicochemical properties.

**Fig. 2:**
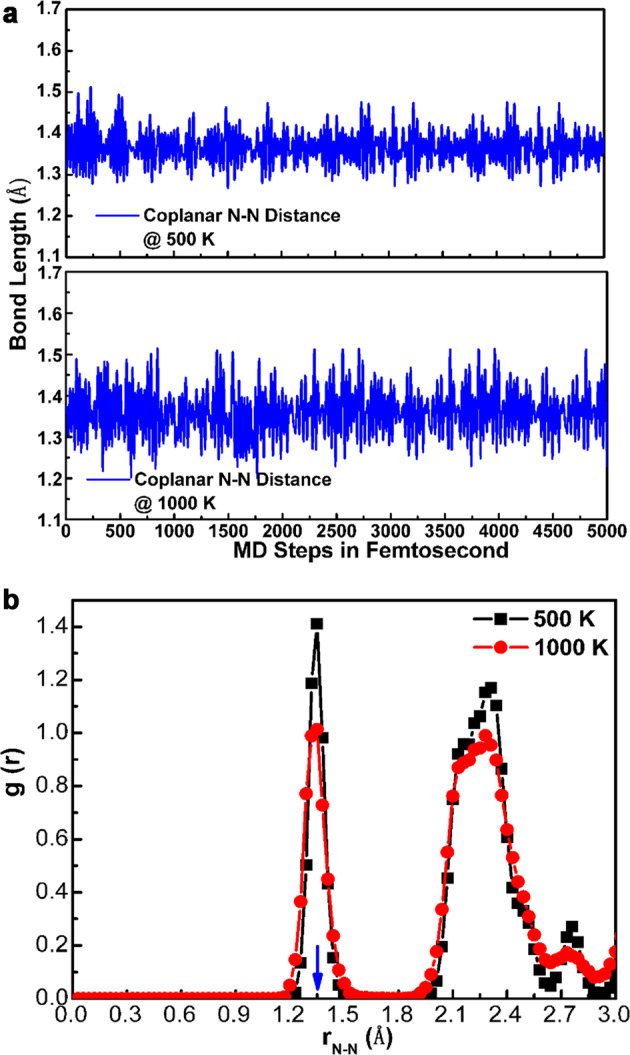
N–N displacements and radial distribution function. **a** N–N bond lengths as a function of time and **b** radial distribution function *g*(r) at 500 and 1000 K under a constant pressure (120 GPa) according to AIMD simulations. The blue arrow denotes the N–N single bond distance according to the previous report.

The possible routes and pressures for synthesizing anti-CdCl_2_ phase are summarized and displayed in Fig. 1d. The N–N single bonds endowed TeN_6_ with superior energy storage properties, which reached 4.79 kJ/g after decomposing into pollution-free nitrogen and the *P*3_1_21-Te phase. The average N–N displacements of the anti-CdCl_2_ phase at 500 and 1000 K after the 50 Ps first-principles molecular dynamics (AIMD) simulations shown in Fig. 2a are still 1.37 Å, which suggests that the structural framework remains basically unchanged and thermodynamically stable. The radial distribution function *g*(r) (Fig. 2b) confirms that the covalent N–N single bonds retained in an isolated peak in cyclo-N_6_ anions were not broken, and the long-range order naturally persists to crystallize even at temperatures up to 1000 K^30^. The phonon dispersion calculation demonstrates mechanical stability as shown in Supplementary Fig. 3. The mechanical natures are identified in Supplementary Table 3. Especially, the bulk modulus of the anti-CdCl_2_ phase is 505 GPa higher than that of diamond 431 GPa^31^, which implies a larger volume compression resistance and covalent bonds equipment. An evidently high value of *C*_33_ (929 GPa) is attached to the crystal, which identifies its remarkable high stiffness along the *c-*axis. The remarkable mechanical properties enable metallic TeN_6_ to better resist external force destruction under extreme conditions.

### Electronic structure and bonding properties

**Fig. 3:**
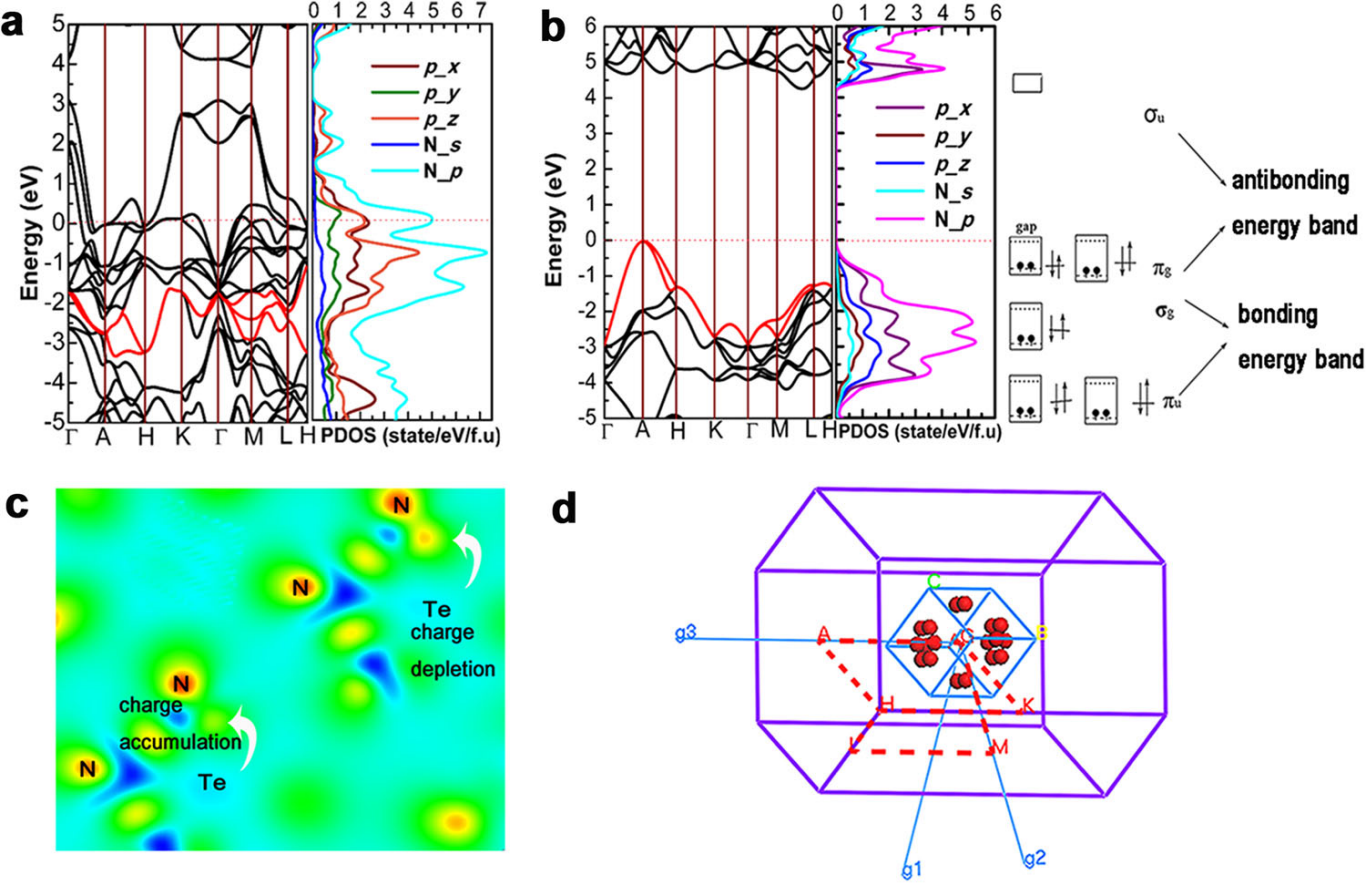
Electronic structure and differential charge density analysis. **a** Electronic band structures and projected density of states (PDOS) with TeN_6_. **b** Planar six-membered N_6_ optimized after removing Te atoms at 120 GPa and its schematic molecular orbital diagram for N atoms. **c** Differential charge density of TeN_6_ projected on the (−0.5 0.6 0.7) plane. **d** Selected high symmetry point and path in the reciprocal lattice space to calculate the energy bands.

**Fig. 4:**
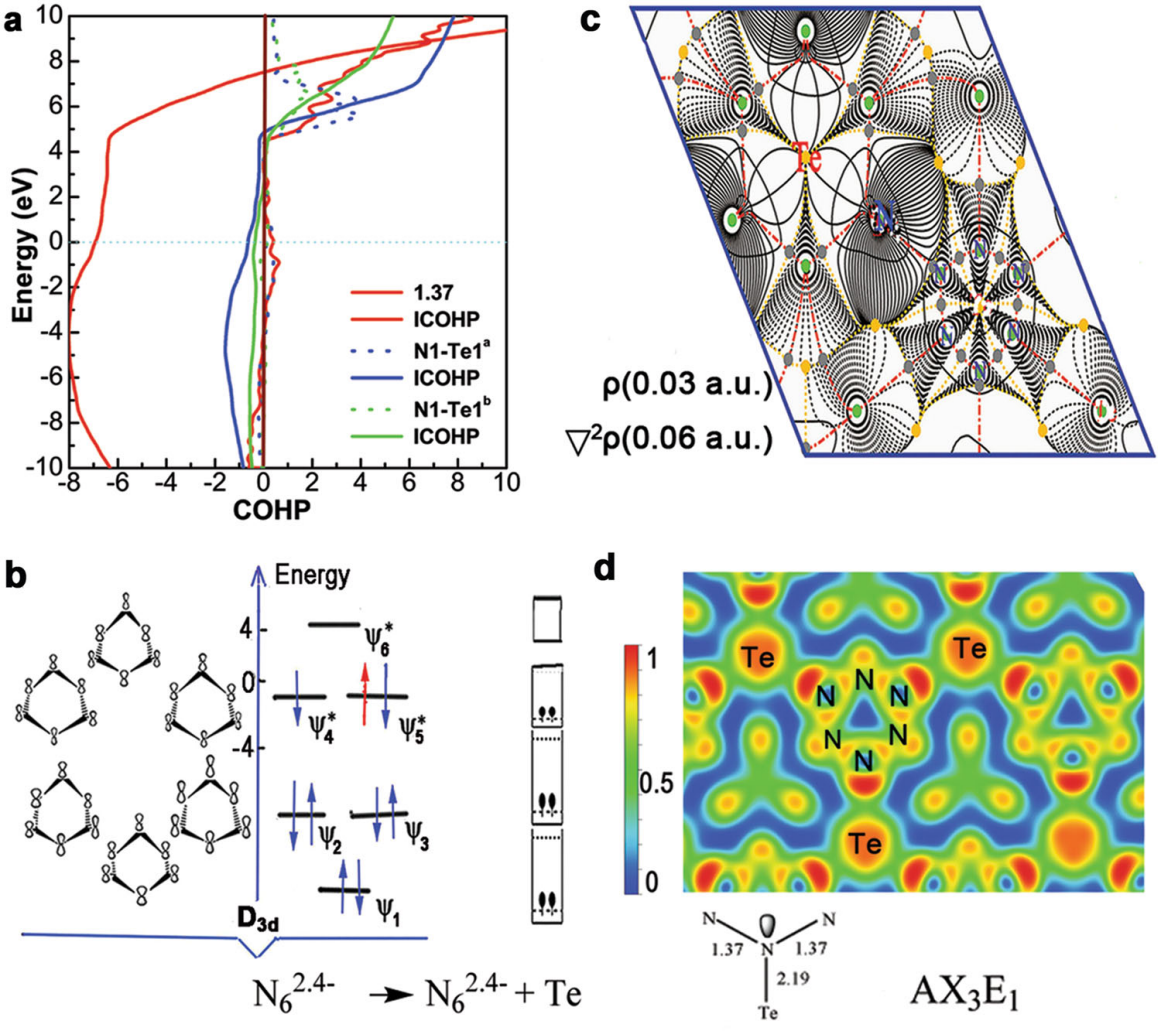
Bonding characteristics under high pressure. **a** Plot of COHP and ICOHP for anti-CdCl_2_-type TeN_6_ at 120 GPa. **b** Simplified correlation diagram of the *p_z* orbitals of cyclo-N_6_ and the schematic orbital overlap of N_6_^2.4−^ MOs, which form because of tellurium. **c** Gradient paths and critical points derived from a QTAIM analysis in the (001) plane. The heavy, dotted, and solid thin lines correspond to the zero, positive, and negative isovalues of Laplacian, respectively. **d** Electron localization functions in the (001) plane and bonding features marked by the VSEPR notation.

Compared with the neutral planar cyclo-N_6_ sub-lattices in the *R-*3*m*-N_6_ phase, the structural deformation induced by tellurium atoms is subject to the Jahn–Teller effect in the anti-CdCl_2_ TeN_6_ phase. A typical symmetry breakage occurs because its geometries with high symmetry produce real or approximate degenerate states. In Fig. 3b, the degeneracy of the *p_x* and *p_y* orbits in the *R-*3*m*-N_6_ phase is obvious near the Fermi level. However, along the K-Г, Г-M, M-L, and L-H directions, the corresponding MOs (red lines) are nondegenerated in the anti-CdCl_2_ TeN_6_ phase and extend in the direction of lower energy. Then, structural distortion inevitably cause changes in the physical properties. The anti-CdCl_2_ TeN_6_ exhibiting metallic property is a sharp contrast to the insulator properties in the *R*-3*m-*N_6_ phase. The dispersion of the N_*p* orbits increases with the strong interaction with tellurium atoms, and a Van Hove singularity is formed near the Fermi level, as shown in Fig. 3a. The consistency of the Te- and N-*p* orbital profiles shown in Supplementary Fig. 4 confirms the *sp*^3^ hybridization. In general, the nonplanar cyclo-N_6_ can inhibit the delocalization effect of *π* electrons, which should be a nonmetallic state^32^. Thus, tellurium atoms play critical roles in bonding and metallic properties for cyclo-N_6_ anions except for causing structural deformation. Consequently, we perform Crystal orbital Hamilton population (COHP) quantitative analysis, as shown in Fig. 4a, to identify the role of tellurium atoms in the bonding interaction with cyclo-N_6_. Intriguingly, the tellurium atom serves as an excellent reductant in ionic crystals and forms an unusual covalent bond with the adjacent cyclo-N_6_ since the integral COHP (ICOHP) value (N1−Te1^a^, nearest neighbor distance) reaches −0.64 eV and the sub-adjacent (N1-Te1^b^, second nearest neighbor distance) is −0.35 eV, which implies an overlap of their electron clouds. Meanwhile, this finding proves that Te atoms (in a formula unit, f.u.) do not completely transfer all the valence electrons (6 *e*) into the antibonding ψ^*^ (N-*p_z*) MOs, as shown in Fig. 4b. This result is also confirmed by the Bader charge analysis, which reveals that the charge from one Te atom to one N_6_ ring is approximately 2.4 *e*. Then, we analyze the metallization process in detail from the perspectives of cyclo-N_6_ and Te MOs. The cyclo-N_6_ unit inherently has six *π* MOs (*P_*z) and *π* electrons: three bonding ψ_1-3_ and three antibonding ψ*_4-6_ MOs. Assuming that no electron transfer occurs, three bonding ψ_1-3_ MOs are occupied, while the higher-energy antibonding MOs ψ_4_* remains vacant and exhibit nonmetallic properties. Considering the interactions with the Te atoms, each ψ MO produces a scattered *π* band along Г-A and K-M in the Brillouin region and induces the band overlap and metallic phenomenon. Meanwhile, the Te 5p electron, as shown in Supplementary Fig. 4, is completely delocalized in this phase, so it has a strong influence on the electronic structures of TeN_6_ except for lowering the Fermi level and plays a relatively significant role in the conducting behavior, which is in strong contrast to Heusler semiconductors^33^.

We further investigated the covalent bond effect of cyclo-N_6_ anions. Through the topological analysis of the coupling electron charge density with Laplacian, the bond critical points (BCPs) derived from a QTAIM^34^ were adopted to further confirm the bonding behavior, as shown in Fig. 4c. In the (001) plane, the solid isovalues of Laplace for its electron density at BCP are negative, which indicates strong covalent interactions. N atoms connect to the nearest neighbors and form bond paths. The nearest Te and N atoms also have negative isovalues of Laplacian; the actual effect between them is due to polar covalent bonds but not entirely of the closed-shell interaction category^35^. Each Te covalently bonds with six N_6_ units, while each N in *sp*^3^ hybridization forms three covalent bonds (one Te and two N atoms), which implies that Te can form abundant delocalized chemical bonds. The three-dimensional structure formed by the effective covalent bonds endows metallic TeN_6_ with higher hardness (H_V_, 24 GPa), as shown in Supplementary Table 3. Meanwhile, the bonding configuration of cyclo-N_6_ changes from AX_2_E_2_ in isolated N_6_ ions to AX_3_E_1_ (Fig. 4d and Supplementary Fig. 5), which effectively restrains the damage caused by the interactions of nonbonding pairs to the system’s stability^36^. In addition to the electrostatic interaction between Te^2.4+^ and N_6_^2.4−^ ions, which reduces the energy by forming strong ionic bonds, the existence of weak covalent bonds leads to the same function since the COHP integral of Te–N leads to a drop of approximately −1 eV/f.u. of the MO energy.

### Chemical insensitivity and detonation performance

**Fig. 5:**
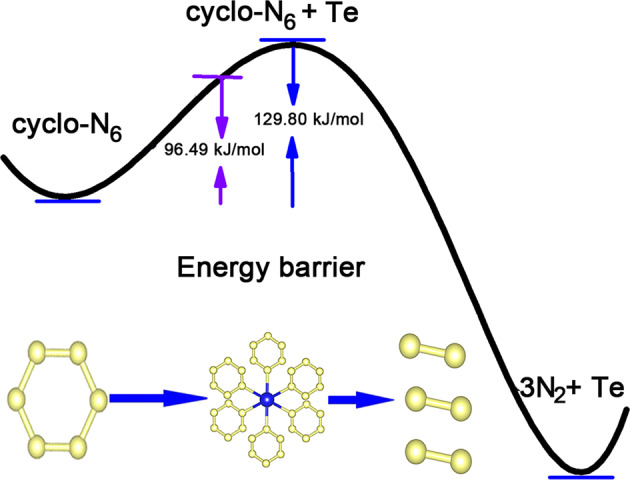
Stabilization of the cyclo-N6 anion by covalent bonds entrapment. Tellurium mixing enhances the insensitivity of the cyclo-N_6_ anion by improving the energy barriers by 129.80 kJ/mol per f.u.; the potential barrier induced by the covalent bond is 96.49 kJ/mol per f.u. relative to the isolated cyclo-N_6_ in the stability effect.

Nitrides are unstable energy-intensive materials and are expected to be highly insensitive, which allows their use in detonation applications^15^. The discovered Te–N covalent bonds led us to investigate their energy barrier. According to the ICOHP calculation of the Te–N bonds, the potential barrier induced by the covalent bond is 96.49 kJ/mol per f.u., as shown in Fig. 5, which accounts for most of the tellurium mixing energy barriers (129.80 kJ/mol), and plays an important role in improving the chemical barrier and insensitivity property. Moreover, the study of the stability of arylpentazoles indicates that the potential barriers of their various compounds are 78–100 kJ/mol, which are lower than the predicted potential barriers of cyclo-N_6_ in our report^37^. As is known, a large pressure is required to overcome the energy barrier (~82.98 kJ/mol) of dinitrogen N_2_ to shape the poly-nitrogen phase during synthesis. A higher barrier induced by covalent bonds can resist the spontaneous decomposition of cyclo-N_6_. In contrast, for the known quasi-planar cyclo-N_6_^2−^ anions in the *C*2/*m*-CsN_3_ phase (Supplementary Fig. 6 and Supplementary Table 1), covalent interactions did not occur between cesium and cyclo-N_6_ anions since all the ICOHP values presented in Supplementary Table 2 are greater than 0. However, the armchair-like cyclo-N_6_ in *h*-WN_6_ has covalent bond characteristics, which shows that armchair-like N_6_ anions have extra stability and prevent the decomposition of the monatomic forms of solid cyclo-N_6_ ions into molecular phases.

The detonation performance estimated by the Kamlet−Jacobs empirical equations is one of the most important indicators of energetic materials^38^. The detonation performances of traditional high-energy-density materials, e.g., TNT and RDX, are shown in Supplementary Table 4, and detailed descriptions are provided in Supplementary Note 1^39^. The cyclo-N_6_ anions that release a large amount of nitrogen are considered environmental friendly clean energetic materials. According to the principle of maximum heat release, the detonation products are determined to be tellurium and nitrogen under ambient conditions. Herein, the gravimetric energy loading density of TeN_6_ was calculated to be approximately 8.16 g/cm^3^. We estimated the detonation velocity (*D*) and detonation pressure (*P*) using decomposition products at ambient pressure. Intriguingly, due to the dual effects of its high-energy density and loading density, its detonation pressure is four times greater than that of traditional TNT and two times greater than that of pentazolate anion in MgN_10_ salt^40,41^.

In summary, we report an armchair-like cyclo-N_6_ anion salt with an inherent single covalent bond though swarm-intelligence structure searches of the TeN_6_ system. The covalent bond modifies the distribution density of local electron clouds and effectively increases the kinetic energy, which is an important factor for metallization in the TeN_6_ structure. More importantly, the armchair-like cyclo-N_6_ anion can be stabilized by additional covalent bond entrapment, which effectively improves the chemical barrier and insensitivity. In addition, the energy density of cyclo-N_6_ anions is higher than that of multitudinous pentazolate and six-membered anions under high pressure in binary nitrides. The simulated detonation performance of the armchair-like cyclo-N_6_ anion salt is much higher than that of traditional TNT/RDX blasting materials. This study is important regarding the insensitivity of cyclo-N_6_ anions and may facilitate high-pressure synthesis.

## Methods

### Structure search

The predicted crystalline phases are based on the global minimization of energy surface merging particle swarm optimization methodology as actualized in the CALYPSO code^42^. The favorite structures of tellurium nitride were predicted at 0, 20, 50, 100, 150, and 200 GPa using the simulation cell, which consisted of 2−4 f.u.

### Electronic structure and total energy calculations

Density functional theory in the Perdew–Burke–Ernzerhof parameterization of the generalized gradient approximation as implemented in the Vienna ab initio simulation (VASP) code was employed for the relaxations^43–45^. Van der Waals (vdW-DF2) interactions were used to correct the structural rationality^46^. The projector-augmented wave method was utilized with the Te and N potentials, where 5*s*^2^5*p*^4^ and 2*s*^2^2*p*^3^ were considered valence electrons. A plane-wave (PW) basis set cutoff of 850 eV and a Monkhorst–Pack *k* meshes spacing of 2*π* × 0.03 Å^−1^ were used; the self-consistent field tolerance was of 0.1 × 10^−5^ eV/atom. The COHP analyses executed in the LOBSTER code^47^ were performed for the TeN_6_ compound to elucidate its bonding information. To provide detailed information, the COHP was calculated based on the PW method and was performed by re-extracting atom-resolved information from the delocalized PW basis sets^48^. Based on counted energy-weighted population of the wave functions between two atomic orbitals, the ICOHP value quantitatively represented the covalent bonding strength. In addition, the phonon calculations were performed using a supercell approach in the finite displacement theory as implemented in the PHONOPY code^49^.

### Molecular dynamics simulation

We also performed a first-principles molecular dynamics simulation to determine the thermal stability of the anti-CdCl_2_ structure via NPT ensembles (N is particle number, P is pressure, and T is temperature). The 84 nitrogen atoms in the super-lattice were used. Molecular dynamics calculations were performed at temperatures of 500 and 1000 K, each of which included 5000 1-fs time steps. Referring to the previous analysis, we generally reached a consensus that the bond lengths of the single bond, double bond, and triple bond were 1.45, 1.25, and 1.10 Å, respectively, under ambient conditions. The N−N single bond distance in *cg*-N is 1.31 Å at 200 GPa^6^, which can guide the assignment of N−N bonds to rationalize the local structural environments with the VSEPR theory.

## Supplementary information

Supplementary Information

## Data Availability

The data supporting this publication are available from the authors on request.
