# Supplementary material for: Formation mechanism of insensitive tellurium hexanitride with armchair-like cyclo-N6 anions
Source: Commun Chem. 2020 Apr 2;3:42. doi: 10.1038/s42004-020-0286-1 (PMC9814709; doi:10.1038/s42004-020-0286-1)
Supplement: Supplementary file 1 — Supplementary Information [file 42004_2020_286_MOESM1_ESM.pdf]

Supporting Information

Formation mechanism of insensitive tellurium hexanitride with armchair-like cyclo-N_6 anions

Zhao Liu†, Da Li†*, Quan Zhuang†, Fubo Tian†, Defang Duan†, Fangfei Li†, Tian Cui‡†*

†*State Key Laboratory of Superhard Materials, Jilin University, Changchun, 130012, People's Republic of China*

‡*School of Physical Science and Technology, Ningbo University, Ningbo, 315211, People's Republic of China*

Corresponding Author

* E-mail: dali@jlu.edu.cn. (D.L.)

* E-mail: cuitian@jlu.edu.cn. (T.C.)

Contents

Supplementary Table 1……..2

Supplementary Table 2……………………...3

Supplementary Table 3……………………...4

Supplementary Table 4……………………...5

Supplementary Note 1……………………..6

Supplementary Figure 1….. ….......................................7

Supplementary Figure 2……………………………………………………………………............8

Supplementary Figure 3……………………………………………………………………….9

Supplementary Figure 4………………………….………………………………………...……10

Supplementary Figure 5………………………..……………………………………..….…11

Supplementary Figure 6…………………………..……………………………………...……12

Supplementary References……….…………………………………………………..….…12

Supplementary Table 1. The unit-cell parameters and atomic positions of the *R*-3*m*-TeN_6 and *R*-3*m*-N_6 phase at 120 GPa, *C2/m*-CsN_3 phase at 120 GPa, respectively.

**Structures**	**Lattice parameters (Å)**	**Atomic coordinates (fractional)**	**Sites**
	a= 5.84	N1 (0.45 0.55 0.23)	18*h*
R-3*m*-TeN_6	b= 5.84		
P= 120 GPa	c= 4.37	Te (0.00 0.00 0.00)	3*a*
	α= 90.00		
	β= 90.00		
	γ= 120.00		
	a= 11.17	N1 (0.15 0.88 0.91)	8*j*
C2/m–CsN_3	b= 5.32	N9 (0.49 0.62 0.72)	8*j*
P = 120 GPa	c= 4.86	N17 (0.43 0.25 0.20)	8*j*
	α= 90.00		
	β= 73.11	Cs1(0.38 0.00 0.73)	4*i*
	γ= 90.00	Cs5(0.77 0.00 0.65)	4*i*

Supplementary Table 2. The ICOHP values of Cs/W and N atoms at different distances in *C*2/*m*-CsN_3/*h*-WN_6 phase at 120/100 GPa, respectively.

	**Distance**	**ICOHP**
**Nearest Cs-N[1]**	2.41 Å	0.58
**Second nearest Cs-N**	2.62 Å	0.08
**Nearest W-N[2]**	2.13 Å	-5.39

Supplementary Table 3. The calculated elastic constants C_{ij} (GPa) and bulk moduli B_0 (GPa), G_0 (GPa), r (G/B) as well as equilibrium H_V (GPa) together with available diamond data and other computational results. The *R*-3*m*-TeN_6 satisfy mechanical Born–Huang criterion. We adopt Chen's model to evaluate Vickers hardness[3].

	C_{11}	C_{33}	C_{44}	C_{12}	C_{13}	C_{66}	B_0	G_0	r	H_v
R-3*m*-TeN_6	829	929	312	309	338	255	505	280	0.27	24
Diamond	1055		564	119			431			96

$C_{11} > C_{12}$, $C_{44} > 0$, $C_{66} > 0$, $(C_{11} - C_{12}) > 0$, $(C_{11} + C_{33} - 2*C_{13}) > 0$, $(C_{11} + 2*C_{12})*C_{33} > 2*C_{13}^2$

Supplementary Table 4. The energy densities (kJ g^{-1}), detonation velocity D (km s^{-1}), detonation pressure P (GPa) and loading density ϱ (g•cm^{-3}) of the phases at ground-state conditions, respectively.

Structures	Pressure	Energy Density	Detonation Velocity (*D*)	Detonation Pressure (*P*)	Loading Density (*ρ*)	Decomposition path at ambient condition
Anti-$CdCl_2$ type TeN_6	> 100 GPa	4.79	10.93	90.18	8.16	$TeN_6 \rightarrow Te + 3N_2$
[4]MgN_{10}	> 12 GPa	3.84	7.16	58.6	2.06	MgN10→1/3[Mg_3N_2+14N_2]
[3]TNT		4.18	7.37	23.46	1.72	
[3]RDX		5.40	8.93	35.31	1.79	

Supplementary Note 1. The Detonation performance estimated by Kamlet−Jacobs empirical equations is one of the most important indicators for energetic materials[5, 6]. The detonation performances of traditional high energy density materials, e.g., TNT and RDX are shown in Table S4. The synthesis of cyclo-N_6 ionic salts releasing a large amount of nitrogen will be considered as an environmentally friendly clean energetic material. According to the principle of maximum heat release, the detonation products are determined to be tellurium and nitrogen at ambient condition. Here, the gravimetric energy loading density for TeN_6 was calculated about 8.16 g/cm^3. The cyclo-N_6 salt has superior energy properties compared to pentazolate anions, while no successful synthesis has been carried out in experiment, the stable armchair-like N_6 salt predicted in our report may be able to achieve in the experiment and further enrich the diversification of poly-nitrogen materials.

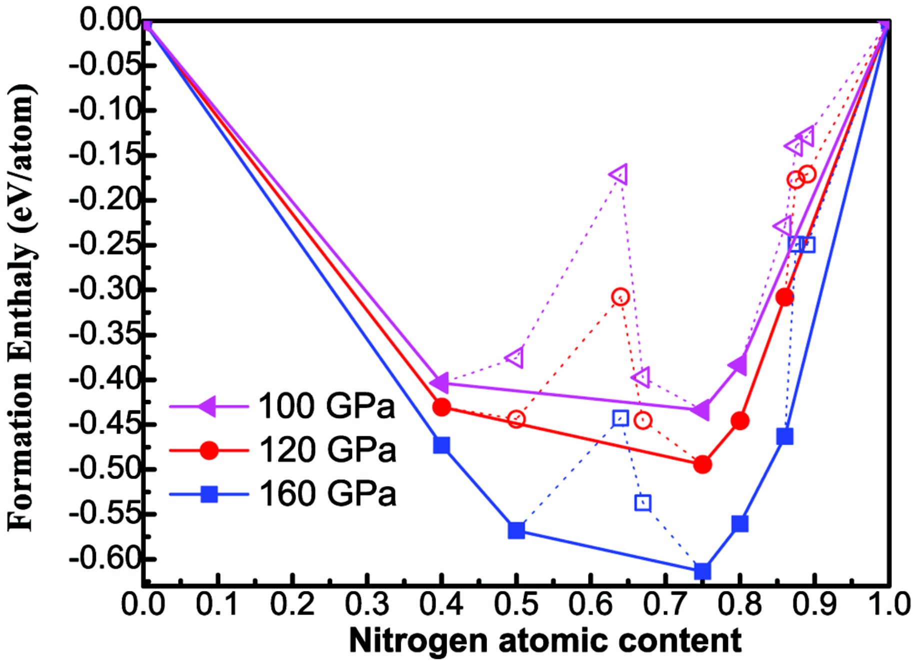

Supplementary Figure 1. The formation enthalpies (eV/atom) of Te − N phases with respect to elemental tellurium and nitrogen solids. The convex hulls at selected pressure are connected by solid lines.

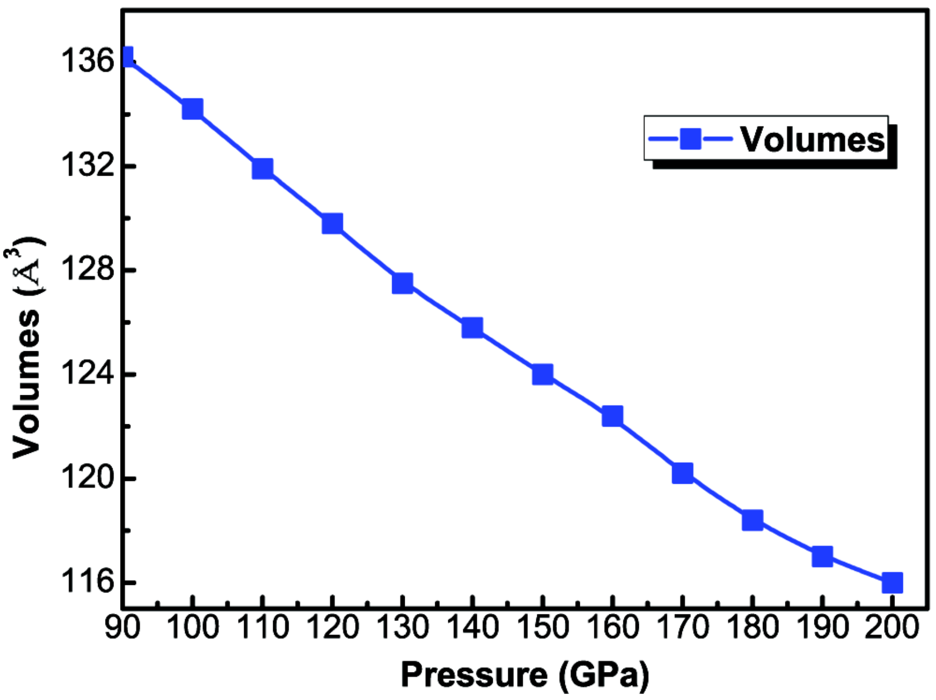

Supplementary Figure 2. The volumes of TeN_6 as a function of pressure.

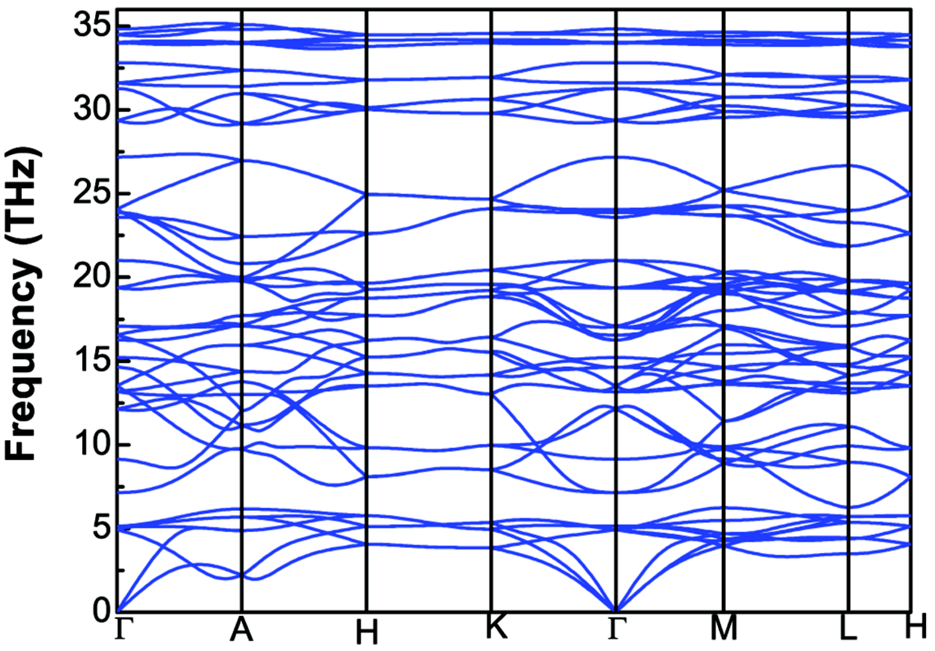

Supplementary Figure 3. Phonon dispersion curve for the *R*-3*m*-TeN_6 phase at 120 GPa.

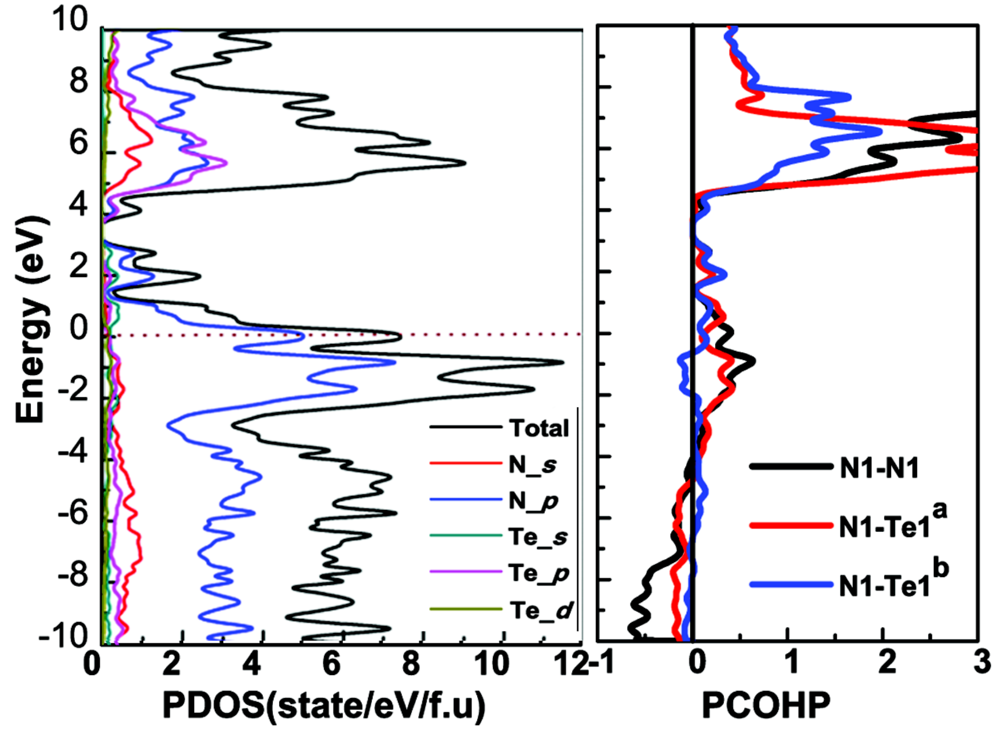

Supplementary Figure 4. Projected densities of states (DOS) and plot of COHP for *R*-3*m*-TeN_6 symmetry at 120 GPa. The negative and positive COHP values denote bonding and anti-bonding interactions, respectively.

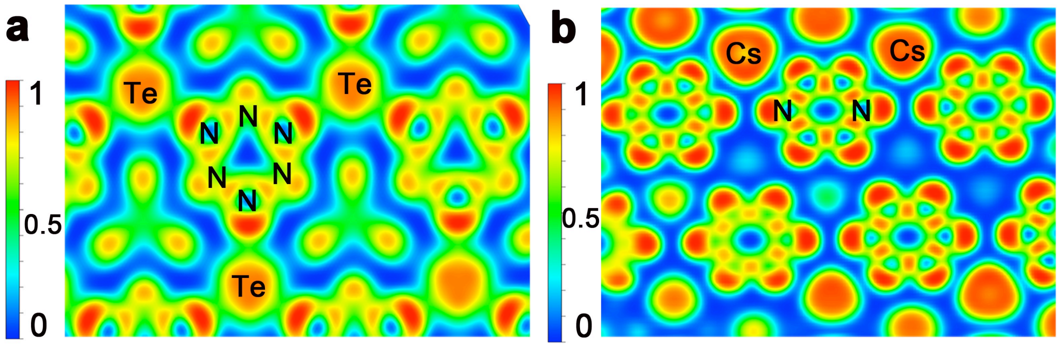

Supplementary Figure 5. The 2D electron localization function slices of **a** *R*-3*m*-TeN_6 and **b** *C*2/*m*–CsN_3 in the (001), (100) planes, respectively. The isosurface of the electron localization function with an isovalue of 0.8.

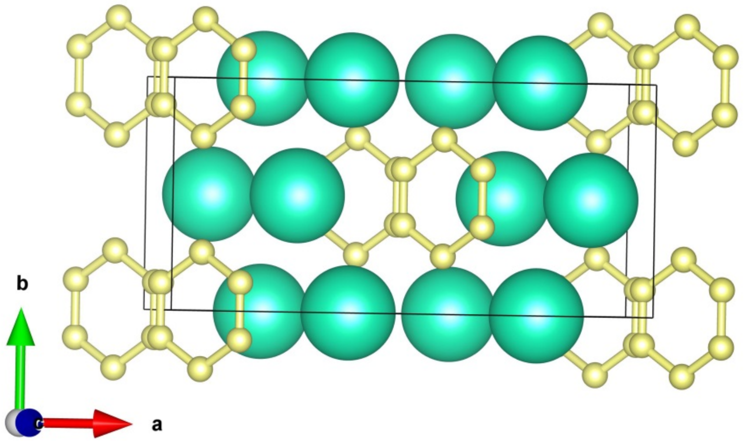

Supplementary Figure 6. The stable structures of *C*2/*m*–CsN_3 phase with N_6^{2-} isolated anion at 120 GPa.

Supplementary References

1. Peng, F., *et al.* Exotic stable cesium polynitrides at high pressure. *Sci. Rep.* **5**, 16902 (2015).
2. Xia, K., *et al.* A novel superhard tungsten nitride predicted by machine-learning accelerated crystal structure search. *Sci. Bull.* **63**, 817-824 (2018).
3. Agrawal, J. P. High Energy Materials: Propellants, Explosives and Pyritechnics; Wiley: Weinheim, (2010).
4. Xia, K., *et al.* Pressure-Stabilized High-Energy-Density Alkaline-Earth-Metal Pentazolate Salts. *J. Phys. Chem. C* **123**, 10205-10211 (2019).
5. Kazandjian, L., Danel, J.F. A Discussion of the Kamlet-Jacobs Formula for the Detonation Pressure. *Propellants Explos Pyrotech* **31**, 20-24 (2006).
6. Kamlet, M.J., Jacobs, S.J. Chemistry of Detonations. I. A Simple Method for Calculating Detonation Properties of C–H–N–O Explosives. *J. Chem. Phys.* **48**, 23-35 (1968).
